# Long-term STED imaging of membrane packing and dynamics by exchangeable polarity-sensitive dyes

**DOI:** 10.1016/j.bpr.2022.100057

**Published:** 2022-04-27

**Authors:** Pablo Carravilla, Anindita Dasgupta, Gaukhar Zhurgenbayeva, Dmytro I. Danylchuk, Andrey S. Klymchenko, Erdinc Sezgin, Christian Eggeling

## Main text

(Biophysical Reports *1*, 100023; December 8, 2021)

An error appears in the second sentence of the Figure 5 legend, which currently reads “(*A*) *xz* GP micrographs of a confocal time-lapse of a GUV made of DOPC:DOPE:DOPS (4:3:3 mol ratio) and a POPC:Chol (2:1 mol ratio) SLB.” The sentence should instead read as follows: “(*A*) *xz* GP micrographs of a confocal time-lapse of a GUV made of POPC:Chol (2:1 mol ratio) and a DOPC:DOPE:DOPS (4:3:3 mol ratio) SLB.”

