# Supplementary material for: Long-term STED imaging of membrane packing and dynamics by exchangeable polarity-sensitive dyes
Source: Biophys Rep (N Y). 2021 Dec 8;1(2):None. doi: 10.1016/j.bpr.2021.100023 (PMC8651516; doi:10.1016/j.bpr.2021.100023)
Supplement: Supplementary file 2 [file mmc9.pdf]

Biophysical Reports

ARTICLE

Long-term STED imaging of membrane packing and dynamics by exchangeable polarity-sensitive dyes

Pablo Carravilla,[1,2,*] Anindita Dasgupta,[1,2] Gaukhar Zhurgenbayeva,[2,3] Dmytro I. Danylchuk,[4] Andrey S. Klymchenko,[4] Erdinc Sezgin,[5,*] and Christian Eggeling[1,2,3,6,7]

[1]Leibniz Institute of Photonic Technology e.V., Jena, Germany; [2]Faculty of Physics and Astronomy, Institute of Applied Optics and Biophysics, Friedrich Schiller University Jena, Jena, Germany; [3]Jena School for Microbial Communication, Friedrich Schiller University Jena, Jena, Germany; [4]Laboratoire de Bioimagerie et Pathologies, UMR 7021 CNRS, Université de Strasbourg, Illkirch, France; [5]Science for Life Laboratory, Department of Women's and Children's Health, Karolinska Institutet, Stockholm, Sweden; [6]Medical Research Council Human Immunology Unit, Weatherall Institute of Molecular Medicine, University of Oxford, Oxford, United Kingdom; and [7]Jena Center for Soft Matter, Jena, Germany

ABSTRACT Understanding the plasma membrane nanoscale organization and dynamics in living cells requires microscopy techniques with high spatial and temporal resolution that permit for long acquisition times and allow for the quantification of membrane biophysical properties, such as lipid ordering. Among the most popular super-resolution techniques, stimulated emission depletion (STED) microscopy offers one of the highest temporal resolutions, ultimately defined by the scanning speed. However, monitoring live processes using STED microscopy is significantly limited by photobleaching, which recently has been circumvented by exchangeable membrane dyes that only temporarily reside in the membrane. Here, we show that NR4A, a polarity-sensitive exchangeable plasma membrane probe based on Nile red, permits the super-resolved quantification of membrane biophysical parameters in real time with high temporal and spatial resolution as well as long acquisition times. The potential of this polarity-sensitive exchangeable dye is showcased by live-cell real-time three-dimensional STED recordings of bleb formation and lipid exchange during membrane fusion as well as by STED-fluorescence correlation spectroscopy experiments for the simultaneous quantification of membrane dynamics and lipid packing that correlate in model and live-cell membranes.

WHY IT MATTERS Stimulated emission depletion super-resolution microscopy can induce photobleaching (i.e., irreversible fluorophore destruction), limiting long-term sample observation. Exchangeable dyes circumvent this problem by only temporarily binding to their target. Here, we used NR4A, an exchangeable dye that also reports on membrane packing, to image model and live-cell plasma membranes. We demonstrate that NR4A enables long-term quantification of membrane biophysical properties, even at the highest stimulated emission depletion resolution, without photobleaching-induced signal loss. NR4A can also be used to simultaneously measure membrane packing and dynamics. By combining the benefits of super-resolution microscopy, fluorescence spectroscopy, and long-term imaging, exchangeable dyes open new possibilities to image dynamic processes with high temporal and spatial resolution, which we demonstrate by imaging lipid exchange during membrane fusion.

INTRODUCTION

Living organisms meticulously adapt their membrane physical and chemical properties (1). These changes occur at different temporal and spatial scales; for example, Example 1: cells can adapt their whole lipidome upon differentiation in a process that takes days (2), Example 2: some enveloped viruses, such as HIV-1, assemble within minutes in submicrometric budding sites with a distinct lipid composition (3,4), Example 3: and lipid molecules can establish millisecond-lived interactions within nanometric trapping sites (5). Fluorescence microscopy is a powerful tool for the study of cell membranes, and by combining it with spectroscopy methods, a number of membrane biophysical parameters can be quantified, such as molecular mobility by fluorescence correlation spectroscopy (FCS) (6), lipid packing using polarity-sensitive dyes in combination with observation of spectral shifts in fluorescence emission (7), or membrane tension by

Submitted June 8, 2021, and accepted for publication September 8, 2021.

*Correspondence: pablo.carravilla@leibniz-ipht.de or erdinc.sezgin@ki.se

Editor: Ulrike Endesfelder.

https://doi.org/10.1016/j.bpr.2021.100023

© 2021 The Authors.

This is an open access article under the CC BY license (http://creativecommons.org/licenses/by/4.0/).

measuring fluorescence lifetime (8). Unfortunately, no fluorescence microscopy technique could so far access the whole range of temporal and spatial scales (i.e., combining super-resolution with long acquisition times and simultaneous readout of biophysical parameters), and new versatile approaches, together with improved and sensitive fluorescent probes, are required to study biological membranes.

Light diffraction limits the spatial resolution of optical microscopes. This was circumvented by super-resolution microscopy (SRM) techniques, which have been extensively applied to membrane research (9). However, SRM introduced new limitations to temporal resolution; for instance, single-molecule localization microscopy (SMLM) (10) and MINFLUX imaging (11) rely on individual molecules emitting photons at different times, and thus for high localization precision, they require prolonged acquisitions in the range of several minutes (12,13). Stimulated emission depletion (STED) microscopy (14) is, in principle, compatible with time-lapse acquisitions because its acquisition speed is limited by the scanning frequency, allowing frame times in the range of a few seconds. Moreover, STED microscopy can be combined with spectroscopic techniques, such as STED-FCS (5), raster image correlation spectroscopy (15), or spectrally resolved imaging (16,17). Unfortunately, although the STED phenomenon is reversible and, in theory, does not induce enhanced dye destruction (but rather a reduction) (18), photobleaching rates are above those of conventional microscopy, especially at high STED laser powers required to achieve the highest spatial resolution (19,20). This higher photobleaching rate seems to be associated with transitions to the more reactive higher excited singlet and triplet states (21,22) through absorption of STED photons (19,20,23). Photobleaching remains one of the major complications of STED microscopy, despite big efforts to avoid it, such as the development of novel photostable dyes (24,25), separation of excitation pulses (26), the use of high scanning rates (27,28), or adaptive illumination modes (29).

An alternative method to circumvent photobleaching is the use of exchangeable dyes. Exchangeable dyes transiently bind to their target and are subsequently removed, thus minimizing the photobleaching probability and eliminating photobleached molecules. Exchangeable dyes are the basis for PAINT (point accumulation for imaging in nanoscale topography) (30), which has been applied for spectral and temporal multiplexing in SMLM (31). Interestingly, the PAINT concept was introduced by exploiting the transient binding of the exchangeable Nile red dye to lipid membranes (30,32). Since then, Nile red has been used in combination with spectrally resolved SMLM for quantitative cell membrane packing imaging (12,33–35). In a recent work, Spahn et al. demonstrated the potential use of exchangeable dyes in STED microscopy by imaging live bacterial and eukaryotic membranes with Nile red over the course of minutes (36). The efficacy of Nile red as an exchangeable dye relies on its polarity-sensitive nature (37); because its quantum yield in aqueous solutions is much lower than in hydrophobic environments (38), only those Nile red molecules bound to membranes are effectively visualized (30).

Polarity-sensitive dyes, also known as solvatochromic dyes, change their emission spectra in response to the polarity of the environment because of the dipolar relaxation effect (39,40). In a membrane context, polarity is directly related to the presence of water molecules at the bilayer interface, which is determined by lipid packing (41) (Fig. 1 *A*). Thus, polarity-sensitive dyes have been extensively used to study the molecular order of membranes and their phase behavior, i.e., their organization into environments of high (liquid-ordered, (Lo)) and low (liquid-disordered (Ld) lipid packing (42). Laurdan was the first polarity-sensitive dye widely used to study cell membranes because of its high sensitivity to packing changes and its homogeneous distribution within ordered and disordered environments. However, high susceptibility to photobleaching, Laurdan's incompatibility with SRM, and an extremely high internalization rate that hinders plasma membrane staining (a limitation shared with Nile red), have motivated researchers to find alternative dyes, such as di-4-ANEPPDHQ (43,44), or new derivatives of Laurdan (45) and Nile red (12,46) to investigate plasma membranes.

To investigate spectral changes in combination with microscopy, in a typical experiment, emitted photons are spectrally separated and collected by two detectors, the lower (blue-shifted) and higher (red-shifted) wavelength signals corresponding to the ordered and disordered channels, respectively. The generalized polarization (GP) ratiometric function (see Materials and methods) (41) is then used to quantify lipid packing (7,47), with higher GP-values corresponding to ordered membranes and lower values to loosely packed lipid environments. In an alternative approach, spectral detectors available in some commercial microscopes can be used to measure the emission spectra of polarity-sensitive dyes in every pixel, combining the advantages of microscopy and spectroscopy (48).

Here, we characterize the spectral properties and STED microscopy performance of NR4A, a recently developed Nile red exchangeable derivative (12) that selectively stains plasma membranes. We found that, because of its enhanced brightness, NR4A shows superior resolution and performance in STED and STED-FCS measurements. Because of its exchangeable nature, NR4A circumvents photobleaching-induced

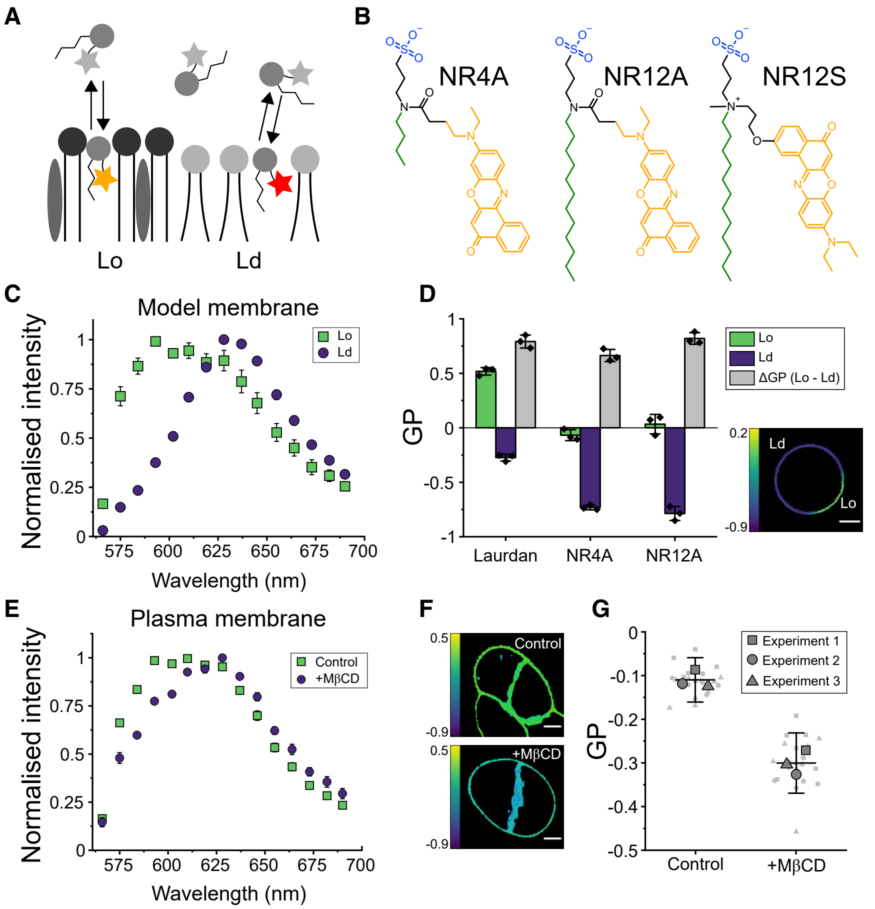

FIGURE 1 The exchangeable dye NR4A can sense lipid packing changes in model and cell plasma membranes. (*A*) Exchangeable dyes transiently partition to biological membranes, where their fluorescence spectrum is dependent on lipid packing. (*B*) Dyes used in this study. Sulfonate groups are marked in blue, alkyl chains are marked in green, and Nile red is marked in orange. Because of its short alkyl chain, NR4A is an exchangeable dye, whereas NR12A irreversibly binds to membranes. NR4A and NR12A were designed based on NR12S. (*C*) The emission spectrum of NR4A shifts in response to lipid packing. Phase-separated GUVs were labeled with 250 nM NR4A and imaged by confocal microscopy using spectral detectors. (*D*) GP analysis of spectral imaging of phase-separated GUVs stained with Laurdan (250 nM), NR4A (250 nM), and NR12A (20 nM). ΔGP is the difference between GP-values measured in Lo and Ld environments. The points represent the mean GP of at least five GUVs in each of three independent experiments. The micrograph shows the GP image of a phase-separated GUV stained with NR4A. (*E*) Spectral imaging of living C2BBe1 cells shows that the NR4A emission spectrum undergoes a red-shift upon cholesterol depletion induced by 20 mM MβCD treatment for 1 h. (*F*) GP images of control (PM, *top*) and MβCD-treated plasma membranes (*bottom*). GP-values were calculated from spectral imaging data. (*G*) Quantification of plasma membrane GP-values shows a significant GP difference upon MβCD treatment. Big symbols are the mean GP-value of at least five cells for each independent experiment, whereas small symbols represent the GP-value of each image. Each symbol (*square*, *triangle*, and *circle*) represents an independent experiment. All measurements were performed at room temperature. In all panels, whiskers are the SD of three independent experiments. Scale bars, 5 μm. Lo, liquid-ordered; Ld, liquid-disordered.

signal loss in STED microscopy experiments and permits long-term super-resolved quantification of plasma membrane biophysical properties, namely lipid packing and dynamics. We showcase possible applications of NR4A by using three-dimensional (3D) STED to image giant plasma membrane vesicle (GPMV) formation and lipid exchange during membrane fusion.

MATERIALS AND METHODS

Dye synthesis

The following fluorescent probes were synthesized according to the previously published procedures: NR4A (12), NR12A (12), and NR12S (46).

Model membrane systems

1,2-dioleoyl-sn-glycero-3-phosphocholine (DOPC), 1,2-dioleoyl-sn-glycero-3-phosphoethanolamine (DOPE), 1,2-dioleoyl-sn-glycero-3-phospho-L-serine (DOPS), 1-palmitoyl-2-oleoyl-glycero-3-phosphocholine (POPC), egg sphingomyelin, and cholesterol were purchased from Avanti Polar Lipids Alabaster, AL). Supported lipid bilayers (SLBs) were formed by spin-coating (49). Glass coverslips (#1.5) were immersed in piranha solution (H_2SO_4:H_2O_2 3:1 vol ratio) for 2 h, followed by five washing steps with abundant distilled H_2O. Clean coverslips were stored in H_2O for up to a week. Lipid mixtures were prepared in chloroform:methanol (2:1 vol ratio) at a concentration of 1 g/L. 25 μL of the lipid mixture were added to a piranha-cleaned coverslip and immediately (within the same second) spread through spin-coating at 3000 rpm for 30 s. The coverslip was mounted in an Attofluor chamber (Thermo Fisher Scientific, Waltham, MA), and the resulting lipid film was hydrated in HEPES-buffered saline (HBS; 150 mM NaCl, 10 mM HEPES (pH 7.0)) and washed thoroughly with HBS five times. SLBs were labeled by adding 10–20 nM NR12A/NR12S or 200 nM NR4A.

Giant unilamellar vesicles (GUVs) were prepared using the electroformation method. Lipid mixtures were prepared at a final concentration of 1 g/L in chloroform. The lipid mixture (5 μL) was spread on two parallel platinum wires and subsequently dried with a gentle argon stream and 5-min vacuum. The wires were dipped in a homemade polytetrafluoroethylene chamber filled with 300 mM sucrose. GUVs were formed by connecting the wires to a function generator and applying a 2 V, 10 Hz of alternating current for 1 h, followed by a 2 V, 2 Hz current for 30 min. GUVs were collected with a 1000-μL plastic pipette tip; to avoid GUVs rupture, the tip diameter was widened by cutting it ~2 cm from the end. GUVs were transferred to a new tube containing

750 μL of HBS and left to sink for 15 min because of the higher sucrose density. The bottom 100 μL of the tube were then collected with a cut tip and transferred to a new tube for labeling by adding 10–20 nM NR12A/NR12S. NR4A labeling was attained by adding 200 nM dye directly to the imaging chamber before GUV addition. Finally, GUVs were transferred to a bovine serum albumin-blocked Ibidi eight-well μ-slide prefilled with 300 μL of HBS. The glass surface was blocked before GUV addition by incubating the wells with 200 μL of 2 g/L bovine serum albumin (Sigma-Aldrich, St. Louis, MO) for 15 min, followed by five washing steps with 400 μL of distilled H_2O.

Large unilamellar vesicles (LUVs) were prepared following the extrusion method. Lipid mixtures in chloroform were dried under an argon stream for 1 min, followed by vacuum drying for 45 min. The resulting lipid films were hydrated in HBS to a final 1 mM concentration, followed by 10 freeze (liquid N_2, 1 min) and thaw (37°C water bath, 3 min) cycles and 30 extrusion cycles through two 400-nm filters (Whatman, Maidstone, UK) using a Mini-Extruder (Avanti Polar Lipids).

Cell lines

C2BBe1 human enterocytes were grown at 37°C and 5% CO_2 in Dulbecco's Modified Eagle's Medium (DMEM) supplemented with 10% fetal bovine serum (FBS), 1% L-glutamine, 0.01 mg/mL holo-transferrin, penicillin, and streptomycin. They were subcultivated at a 1/10 ratio every 4 days and kept until passage 20. Cells were seeded on collagen I-coated glass coverslips at a final concentration of 1×10^5 cells/mL in six-well plates. Coverslips were coated with collagen I at a final concentration of 10 μg/mL, incubated 2 h at room temperature in the dark, and subsequently dried at 37°C. Ptk2 potoroo kidney cells were grown at 37°C and 5% CO_2 in DMEM supplemented with 10% FBS, 1% L-glutamine, penicillin, and streptomycin. They were subcultivated at a 1/3 ratio every 3 days and kept for 30 passages. CHO-K1 Chinese hamster ovary cells were grown at 37°C and 5% CO_2 in DMEM-F12 supplemented with 10% FBS. CHO-K1 cells were subcultured at a 1/8 ratio twice per week and kept for 30 passages.

For imaging experiments, cells were grown in 25-mm coverslips for 2 days. Coverslips were mounted on Attofluor chambers, washed twice with Leibovitz's L-15 medium (Thermo Fisher Scientific), and afterwards, the corresponding dye was added. In cholesterol-depletion experiments, C2BBe1 cells were treated with freshly prepared 20 mM methyl-β-cyclodextrin (MβCD; or medium in the case of control cells) and incubated for 1 h at 37°C/5% CO_2. Finally, cells were washed twice with Leibovitz's L-15 medium, labeled with 20–50 nM NR12A or 200–500 nM NR4A, and subsequently imaged.

Spectral imaging and GP calculation

Spectral images were acquired using a Zeiss 880 confocal laser scanning microscope equipped with a C-APOCHROMAT 40×/1.2 W Korr FCS objective lens (Zeiss, Oberkochen, Germany). To avoid photoselection, circular polarization was generated by introducing a phase retarder in the DIC slider of the objective lens. Laurdan (Thermo Fisher Scientific) and NR4A/NR12A were excited using 405- and 561-nm laser lines, respectively, with an excitation power of 10 μW at the sample plane. Emitted photons were descanned, passed through a 1.0-airy unit pinhole, and collected by a 32-spectral channel GaAsP detector with an 800-V gain. Each spectral channel consisted of an 8.9-nm window. Pixel size was set to 100 nm, and the dwell time was set to 2 μs. Images were analyzed using a macro (https://doi.org/10.5281/zenodo.5110173) in Fiji (50). To calculate emission spectra, an intensity threshold was applied to the images to analyze only pixels corresponding to membranes. In the case of phase-separated GUVs, each phase was analyzed separately. For every image, the average intensity signal was then calculated for each channel. To quantify lipid packing, the GP-value for each pixel was calculated using the GP function (41)

$$GP = \frac{I_b - I_r}{I_b + I_r},$$

where I_b and I_r are the intensity recorded at 435 and 510 nm for Laurdan and 575 and 640 nm for NR4A/NR12A. GP-values were calculated using the GP Plugin (48) for Fiji, setting "Noise tolerance" to 0.8 and "Threshold" to 40 (this value was slightly modified if the regions of interest were filtered out in the GP image). The parameters "Sample wavelength low" and "Sample wavelength high" were set to *Ib* and *Ir* wavelengths, respectively. The spectra were not fitted to any model. In the case of two-channel images acquired with STED microscopes, an intensity threshold was applied to the sum of both channel images. Automatic thresholding by Fiji usually yielded satisfactory results, and the threshold was only slightly modified, usually being 20–30% of the maximal intensity. GP-values for each pixel were then calculated using the GP function, where I_b and I_r are the intensities recorded at the ordered (580–630 nm) and disordered (650–700 nm) channels. Mean values for each independent experiment were considered as the mean GP-value of at least five images. Graphical representation in Figs. 1 *G* and 4 *A* is based on a previous report (51). For FCS and STED-FCS GP measurements, I_b and I_r are the average photon counts on each channel.

STED imaging

STED images were acquired in a custom Abberior Expert Line laser scanning STED microscope using an UPlanSApo 60×/1.2 water immersion objective lens equipped with a correction collar (Olympus, Tokyo, Japan). Resolution strongly depended on the right adjustment of the collar, especially in 3D-STED mode. NR4A/NR12A were excited by a 561-nm pulsed diode laser PDL-T 561 (Abberior Instruments, Gottingen, Germany) with an excitation power of 10 μW at the sample plane. Fluorescence was inhibited by a 775-nm PFL-40-3000-775-B1R 40 MHz pulsed laser (MPB Communications, Pointe-Claire, Canada). The STED beam power at the sample plane for each experiment is specified in figure legends. The beam shape for two-dimensional (2D; doughnut) or 3D depletion (bottle shape) was created using a spatial light modulator (SLM) (Fig. S4 *B*). To align the STED beam on top of the excitation PSF, four-color TetraSpeck 100-nm microspheres (Thermo Fisher Scientific) were used as a reference. The STED beam position was corrected with respect to the confocal signal by adjusting the grating of the SLM. The orientation of the depletion beam was fine-tuned by adjusting the SLM offset. Emitted photons were collected through the objective lens, descanned, passed through a 1.0-airy unit pinhole, and finally collected by single-photon counting SPCM-AQRH-14-TR avalanche photodiodes (Excelitas Technologies, Waltham, MA) equipped with appropriate filters (580–630 and 650–700 nm). Pixel size was 40 nm in all dimensions, and pixel dwell time was 10 μs. Each line was scanned three times, and the collected photon intensity signal integrated.

To calculate the effect of NR4A and NR12 photobleaching in STED imaging, a 10×10 μm^2 area of the plasma membrane of Ptk2 cells was imaged using a 250-mW 775-nm laser power. Images were acquired every 10 s for 15 min. The intensity in a 2×2 μm^2 was quantified, the background signal (as measured by the intensity in a 2×2 μm^2 area devoid of cell signal) was subtracted, and the result was normalized to the intensity of the first frame. For each condition, three cells were measured in each independent experiment.

Internalization was quantified in confocal mode by acquiring 1 frame/min for 1 h. The mean pixel intensity in cell areas that could unequivocally be identified as plasma membrane, cytosol, or nucleus

was measured in Fiji by manually selecting them using the "Polygon Selection" tool and the "Measure" function (50). Intensities were normalized to that measured for the plasma membrane. GPMV formation was induced by dithiothreitol (DTT)/paraformaldehyde (PFA) treatment of CHO cells (52). Cells were grown on glass coverslips to a confluence of ca 75%. Cells were washed twice with GPMV buffer (2 mM $CaCl_2$, 150 mM NaCl, 10 mM HEPES (pH 7.4)), and labeled and imaged in GPMV buffer. During image acquisition, 20 mM DTT and 0.7% PFA were added. Model membrane fusion was induced by adding 10 mM $CaCl_2$ to the SLB and GUV/LUV-containing chamber in HBS during image acquisition.

FCS and STED-FCS

FCS data were acquired at an Abberior STEDYCON STED microscope mounted on a Zeiss Axio Observer Z1 inverted body, equipped with an α Plan-Apochromat 100×/1.46 oil immersion objective lens (Zeiss). Samples were excited using a 40-MHz pulsed 561-nm laser and emission stimulated by a pulsed 775-nm laser, with a repetition rate of 40 MHz. Emitted photons were collected by the objective lens, descanned, and recorded by avalanche photodiodes with 580- to 625- and 650- to 700-nm filters corresponding to the ordered and disordered channels, respectively. The signal from the APDs was cloned and sent to a Flex02-08D/C correlator card (Correlator.com). For each STED laser power, SLBs were first focused at the plane showing the highest intensity and then fine-focused (100–400 nm) to obtain maximal amplitude in FCS recordings. The focus position was adjusted after each measurement to fulfill these criteria because small drifts of the sample would induce considerable amplitude changes in FCS curves, especially at high STED powers. FCS and STED-FCS data were acquired for 10–20 s, and for each spot and laser power, at least five measurements were performed in each independent experiment. The obtained FCS curves were fitted using the FoCuS-point software (53) to a 2D Brownian diffusion model:

$$G_{\tau} = \frac{1}{N}\left(1 + \left(\frac{\tau}{\tau_D}\right)^{\alpha}\right)^{-1},$$

where N is the average number of fluorescent particles in the focal volume, τ_D is the average transient time, and α the anomaly parameter. In all cases, α was close to 1 [0.97–1.03], except for STED-FCS recordings at STED laser powers >100 mW, in which α = [0.80–1.00]. To quantify the resolution at different STED powers, DOPC SLBs were measured, and the apparent spot size was calculated using the following equation (49):

$$\omega_0 = 290\text{ nm}\sqrt{\frac{\tau_{D(STED)}}{\tau_{D(conf)}}},$$

where ω_0 is the $1/e^2$ radius of the Gaussian beam, 290 nm is ω_0 in confocal mode, and $\tau_{D(STED)}$ and $\tau_{D(conf)}$ are the transit times at a given STED power and in confocal mode, respectively. Counts per molecule were calculated from FCS experiments by dividing the average count rate of the measurement by the average number of molecules in the observation spot (N). Diffusion coefficients (D) were calculated from τ_D and ω_0 as follows:

$$D = \frac{{\omega_0}^2}{4\tau_D}.$$

For the simultaneous measurement of GP and D, diffusion times from the higher wavelength channel were considered.

RESULTS

NR4A spectral imaging can resolve distinct lipid compositions in model and cell membranes

NR4A was recently developed by linking Nile red (Fig. 1 *B*, *orange*) to a plasma membrane targeting moiety that included an anionic sulfonate head (Fig. 1 *B*, *blue*) and a four-carbon alkyl chain (Fig. 1 *B*, *green*). The sulfonate group decreased cell permeability by decreasing the flip-flop rate through the membrane (12,45), whereas the short alkyl chain permitted transient binding to lipid bilayers (12). NR12A, a Nile-red-based analog of NR4A but with a 12-carbon alkyl chain (Fig. 1 *B*), showed no exchangeable behavior (12) and was used as a control in our experiments. NR4A and NR12A were designed based on NR12S (Fig. 1 *B*), a Nile-red-based plasma membrane dye containing a phenolic oxygen, which was hypothesized to cause reduced photostability (12).

To investigate the sensitivity of NR4A to lipid packing changes, we prepared phase-separated GUVs made of DOPC, sphingomyelin and cholesterol (2:2:1 mol ratio), and stained them with NR4A. Confocal spectral imaging revealed a clear red shift of the emission spectrum in Ld environments as compared with sphingomyelin- and cholesterol-rich Lo environments, with the emission maximal shifting by ~35 nm (Fig. 1 *C*), as it did for NR12A (Fig. S1 *A*). To assess whether NR4A's exchangeable nature influenced its sensitivity to lipid packing, we then quantified the lipid packing resolution as measured by the GP difference (ΔGP) between Lo and Ld environments in phase-separated GUVs (Fig. 1 *D*) and compared it with Laurdan, a good standard for sensitivity. NR4A showed a ΔGP (0.66 ± 0.06) comparable with that of NR12A (0.82 ± 0.05) or Laurdan (0.79 ± 0.06). These results were independent of the excitation wavelength (Fig. S1, *C–E*).

Spectral imaging experiments showed that NR4A is, besides hardly internalizing into the cell (as further outlined later), also sensitive to lipid changes in the plasma membrane of living cells, as exemplified by cholesterol depletion by MβCD treatment of living epithelial C2BBe1 cells (Fig. 1 *E*). A comparable red shift was measured for NR12A (Fig. S1 *B*). Using spectral imaging data for calculation of GP images (48), for which the GP-value for each pixel is represented in a color-coded scale (Fig. 1 *F*), we also observed a significant change in plasma membrane packing (Fig. 1 *G*).

These results demonstrate that NR4A, in line with the earlier report (12), is a polarity-sensitive membrane dye that can resolve distinct lipid packing environments in model and cell membranes, with a performance comparable to that of well-established polarity-sensitive dyes, such as Laurdan.

NR4A enables prolonged time-lapse STED acquisitions of live-cell plasma membranes

To assess whether the exchangeable nature of NR4A could circumvent photobleaching-induced signal loss in STED microscopy measurements, we stained live Ptk2 epithelial cell plasma membranes with NR4A and its nonexchangeable counterpart NR12A. Quantification of the mean intensity recorded upon STED imaging showed a constant signal for NR4A, whereas NR12A was readily photobleached (Fig. 2, *A and B*; Video S1). In some of the cells, the NR4A signal increased around 10% (Fig. 2 *A*), which we ascribe to a small pool of internalized dyes (Fig. 2 *D*). The NR4A intensity was constant for longer than 15 min; however, because of phototoxic effects induced by the 250-mW STED laser, signs of cell death could be observed short around 5 min, as represented by the appearance of bright clusters and membrane protrusions, which were not detected in the absence of STED illumination, even for 1 h (Video S2).

Previously, 90- to 100-nm resolution was obtained using NR12S with STED imaging (16). In line with the reported increased brightness and photostability of NR4A and NR12A as compared with NR12S (12), NR12A showed a significantly higher raw intensity in STED time-lapse acquisitions of Ptk2 cell membranes under the same imaging conditions and concentration compared with NR12S (Fig. S2 *A*). Thus, even if NR12A is readily photobleached in STED experiments, it still shows superior performance compared with NR12S. Importantly, lipid packing quantification, showed that the measured GP increased upon NR12A photobleaching, whereas it remained constant for NR4A (Fig. S2 *B*). This phenomenon can presumably be caused by photobleached dyes reacting with surrounding lipids (54,55), which might be prevented in the case of NR4A because of transient binding and thus lower reaction probability.

NR4A and NR12A specifically label plasma membranes and show reduced internalization

Another important limitation when studying plasma membranes is dye internalization. This problem is aggravated when imaging polarity-sensitive dyes because the molecular order of inner organelle membranes is significantly lower than that of the plasma membrane (56), thus dye internalization induces a change in the emission spectrum. This is the case for Nile red, which can be exploited to image inner organelle membranes (36) but might report an underestimated packing value when studying plasma membranes. NR4A, NR12A, and NR12S were designed to prevent dye internalization by including anionic sulfonate or zwitterionic groups (12,46). Sulfonate

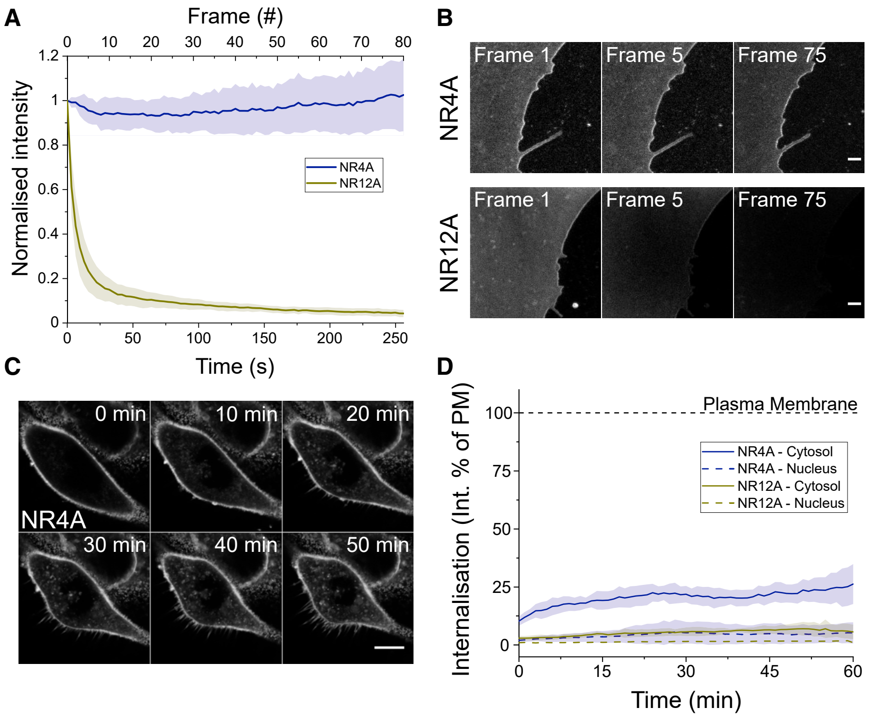

FIGURE 2 STED imaging of live-cell plasma membranes without signal loss. (*A*) NR4A shows a constant intensity signal during super-resolution STED imaging of Ptk2 plasma membranes over the span of minutes. The NR12A signal decreased because of photobleaching induced by the STED laser. The STED laser power was 250 mW at the sample plane. Normalized intensity is the mean intensity in a $2 \times 2\ \mu m^2$ area divided by the initial mean intensity. The colored area corresponds to the SD of three independent experiments. Three cells per condition were measured in each independent experiment. (*B*) Micrographs of STED images of Ptk2 cell plasma membranes illustrating the absence of photobleaching of NR4A as compared with NR12A. Scale bars, 1 μm. (*C*) NR4A selectively stains live-cell plasma membranes. Micrographs of C2BBe1 cells upon incubation with NR4A acquired in confocal mode. (*D*) Quantification of NR4A and NR12A internalization. Internalization is measured as the intensity in regions of interest that could unequivocally be identified as the cytosol or the nucleus divided by the intensity signal recorded at the plasma membrane. The colored area corresponds to the SD of three independent experiments. Dashed line marks 100%, which corresponds to the plasma membrane signal. All measurements were performed at room temperature.

(NR4A, NR12A) and zwitterionic (NR12S) groups reduce transmembrane translocations (flip-flop) of membrane dyes, keeping them in the outer leaflet of plasma membranes (45,46). To measure NR4A internalization, we stained C2BBe1 human epithelial cells and quantified the intensity recorded in three distinct cell regions, namely the plasma membrane, the cytosol, and the nucleus. Even after 50 min, most of the NR4A signal was detected at plasma membranes (Fig. 2, *C and D*; Video S2). However, the cytosolic signal of NR4A was significantly higher than that of NR12A (Fig. 2 *D*), which almost exclusively remained in the plasma membrane for the whole duration of the experiment (Fig. S2 *C*; Video S3). We hypothesize that the highly concentrated unbound pool of NR4A in the extracellular medium could be encapsulated and transported through endocytosis inside the cell, where it would partition to inner membranes.

Our results show that NR4A can be used to perform time-lapse imaging and quantification of lipid packing of live cells using super-resolution STED microscopy, circumventing photobleaching-induced signal loss. In combination with selective plasma membrane staining, this makes NR4A an appropriate dye for time-lapse STED studies. We argue that, although NR12A suffers from photobleaching, it can still be used for experiments in which continuous illumination or high STED powers are not required. Among the advantages of NR12A are its higher selectivity for plasma membranes (Fig. 2 *D*) and the need to use significantly lower concentrations (10–20 nM) as compared with NR4A (250–500 nM) because of NR12A's strict partitioning to membranes (12).

STED-FCS measurements show superior resolution of NR4A and NR12A compared with NR12S

STED microscopy resolution is determined by the photophysics of the imaged fluorophore, thus it has to be measured for each dye independently. Accurately determining the resolution of STED microscopy can be challenging, especially when imaging continuous structures, such as membranes, for which the distance between two dyes continuously changes. STED-FCS constitutes an alternative approach to accurately quantify resolution (49). In an FCS experiment, the average time a molecule takes to cross the confocal volume is measured, thus permitting the calculation of its diffusion coefficient. By measuring the transition time at different STED laser powers on an SLB, we can calculate the volume of the observation spot for each STED power (49). Because an SLB is a 2D structure, this approach reports on the size of the observation spot in the *xy* dimensions (ω_0, see Materials and methods for more details).

To quantify the resolution offered by NR4A, we performed STED-FCS measurements of DOPC SLBs. NR4A and NR12A showed a maximal resolution of 80.4 ± 1.4 and 77.1 ± 1.4 nm, respectively, at 250-mW STED laser power (Fig. 3 *A*). This resolution

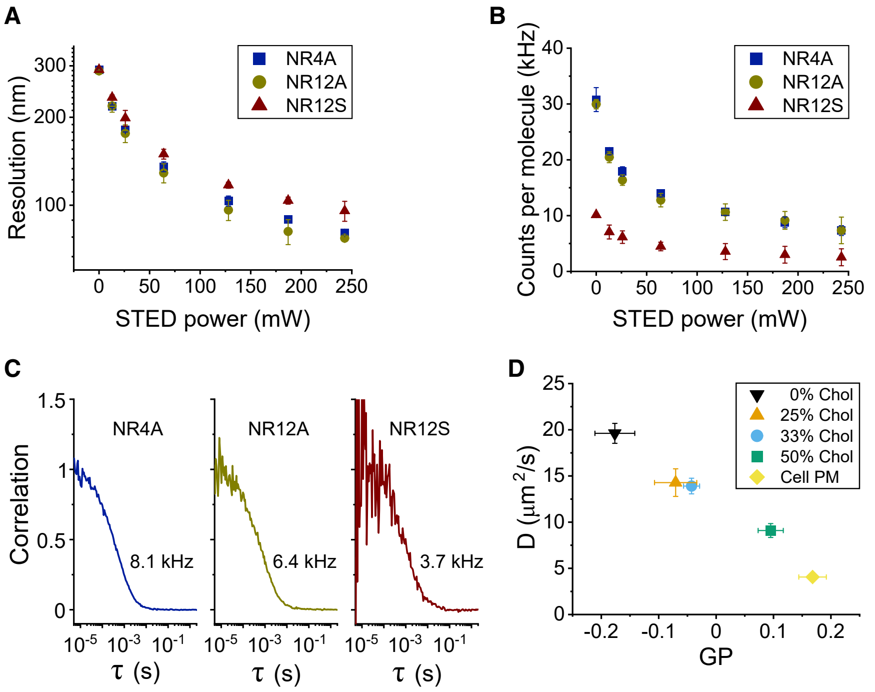

FIGURE 3 STED-FCS measurements of membrane dynamics and packing. (*A*) NR4A and NR12A offer higher resolution than NR12S as quantified by STED-FCS measurements on DOPC SLBs. (*B*) NR4A and NR12A also show superior brightness at all measured resolutions. (*C*) Exemplary STED-FCS curves obtained on DOPC:cholesterol (1:1 mol ratio) SLBs at 250-mW STED laser power. The average counts per molecule of those measurements are indicated. (*D*) Simultaneous quantification of the diffusion coefficient of NR4A and its surrounding lipid packing show a negative correlation between packing and dynamics, as measured at 25-mW STED power in cholesterol-containing SLBs and in the plasma membrane of live Ptk2 cells. The observed anticorrelation is maintained independent of the STED power (Fig. S3 *B*). All measurements were performed at room temperature. In all panels, whiskers are the SD of at least three independent measurements (at least five acquisitions per condition per independent measurement).

is significantly higher than that obtained for NR12S under the same imaging conditions (95.6 $\pm$ 7.4 nm). The major difference between NR4A/NR12A and the original NR12S is the reverse orientation of Nile red in the bilayer and the lack of a phenolic oxygen in the fluorophore of NR4A/NR12A (Fig. 1 *B*), which was hypothesized to cause lower photostability (12). To quantify the effect of this modification in molecular brightness, we measured the counts per molecule of the three dyes at different STED powers using STED-FCS (Fig. 3 *B*). As expected, NR4A and NR12A showed a comparable brightness, approximately three times higher than that of NR12S, which could account for the observed resolution difference. Moreover, STED-FCS curves obtained for NR4A/NR12A showed significantly less noise compared with those measured with NR12S (Fig. 3 *C*), as expected for brighter molecules.

To simultaneously measure membrane dynamics and packing (57,58), we combined STED-FCS acquisitions with GP analysis. For that, we prepared SLBs containing increasing amounts of cholesterol and measured the intensity fluctuations of NR4A in two different spectral channels (580–625 and 650–700 nm) at different resolutions (i.e., STED laser powers). We measured the diffusion coefficient of NR4A using FCS and quantified lipid packing by calculating the GP function from the average photon count measured in each channel. We observed a linear anticorrelation between lipid packing and diffusion at different cholesterol concentrations (Fig. 3 *D*) that was independent of the observation spot size (Fig. S3 *B*). The observed anticorrelation was maintained even when including data acquired on the plasma membrane of live Ptk2 epithelial cells (Fig. 3 *D*). It must be noted that GP-values strongly depend on the STED laser power (16) (Fig. S3 *A*), although the GP resolution (dynamic range) was maintained at all measured STED powers. Thus, STED-GP-values can only be compared with those obtained under the same imaging conditions (e.g., the same STED power and dye concentration).

Altogether, these results show that, due to their increased brightness, NR4A and NR12A offer superior resolution and performance when compared with the previous generation counterpart NR12S in STED, FCS, and STED-FCS experiments. Despite only transiently partitioning to membranes, the binding time for NR4A is longer than the time it takes to cross the observation spot ($\sim$2 ms in cell plasma membranes), making it compatible with FCS measurements because the 2D Brownian diffusion model (see Materials and methods) fitted the correlation data equally as well in the case of the exchangeable NR4A and nonexchangeable NR12A as determined by the residuals of the fitting (Fig. S3 *C*). Moreover, the diffusion time measured for NR4A linearly decreased with cholesterol content independently of the resolution (i.e., STED laser power (Fig. S3 *B*)). Two-channel FCS acquisitions permit the simultaneous calculation of diffusion coefficients and lipid packing by means of the GP function. By employing this approach, we found that the lipid packing and bilayer dynamics anticorrelate in model and live-cell membranes, suggesting that the slow diffusion coefficients commonly measured in plasma membranes can be ascribed to the high degree of lipid packing of this structure, which arises from its distinct lipid composition (56), as previously suggested for highly packed viral membranes (59).

Live 3D-STED imaging of cell membranes

We then proceeded to assess whether NR4A could distinguish lipid packing changes in live cells using STED microscopy. Because our current STED setups were not equipped with spectral detectors, we performed classic two-channel acquisitions using emission filters typically found in STED setups (580–630 nm for the ordered channel and 650–700 nm for the disordered channel). We found that this combination of filters offered a high GP resolution that permitted distinguishing control and cholesterol-depleted (MβCD-treated) plasma membranes at high STED powers (Fig. 4 *A*) and in confocal mode (Fig. S4 *A*).

In 3D-STED, the resolution is increased in three dimensions (*xyz*) by using a “bottle-shaped” depletion scheme instead of the classic “doughnut-shaped” beam (Fig. S4 *B*) (60). 3D-STED was recently shown to be a powerful tool to investigate living membranes (17). Here, we wanted to prove the potential of combining 3D-STED and exchangeable dyes for membrane imaging. 3D-STED microscopy of artificial membranes stained with NR4A clearly showed the increase in lateral and axial resolution (Fig. 4 *B*). As a model to investigate membrane changes in real time, we induced GPMV formation (i.e., plasma membrane vesiculation) with DTT and PFA (52). Plasma membrane vesiculation could be readily observed in CHO cells (Fig. 4 *C*; Video S4) and epithelial human 293T cells (Fig. S4 *C*). The improved axial resolution offered by 3D-STED permitted the visualization of vesiculation and swelling of inner organelles (Fig. 4 *C*). Unfortunately, DTT/PFA treatment induced permeabilization of plasma membranes to NR4A, as demonstrated by the immediate staining of inner membranes (Fig. 4 *C*), whereas untreated cells showed a significantly lower cytosolic signal (Fig. S4 *D*). GPMVs have been shown to be permeable to small solutes; however, cell plasma membranes remain impermeable during GPMV formation (61). In line with these results,

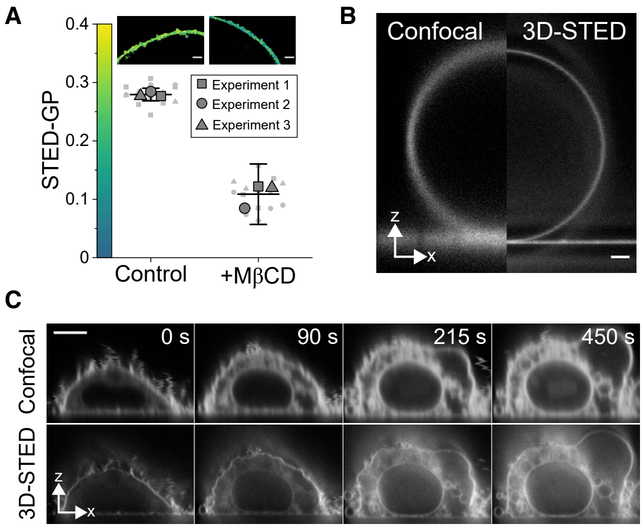

FIGURE 4 3D-STED visualization of live-cell plasma membranes. (*A*) STED GP quantification of cholesterol-depletion-induced packing changes in C2BBe1 cells. STED laser power was 250 mW. Each big symbol is the mean GP-value of each independent experiment (at least five cells), whereas small symbols represent the GP-value of each image. Symbols (*square*, *triangle*, and *circle*) represent independent experiments. Lines represent the mean, and whiskers represent the SD of three independent experiments. (*B*) Confocal and 3D-STED microscopy micrograph of a DOPC GUV labeled with 200 nM NR4A. Scale bars, 1 μm. (*C*) GPMV formation induced by DTT/PFA treatment of CHO cell plasma membranes as visualized by NR4A confocal (*top*) and 3D-STED (*bottom*) imaging. Because of DTT/PFA-induced membrane disruption, NR4A can diffuse inside the cell. NR4A concentration was 500 nM. Scale bars, 5 μm. All measurements were performed at room temperature.

DTT/PFA treatment did not induce plasma membrane permeabilization to hydrophilic solutes, whereas NR4A got internalized (Fig. S4 *E*), hampering a detailed plasma membrane packing analysis during GPMV formation.

Super-resolved live monitoring of lipid packing during membrane fusion

In vitro studies of membrane remodeling processes are among the main applications of polarity-sensitive dyes. Real-time monitoring of such phenomena has so far been hampered by photobleaching. Taking advantage of the exchangeable nature of NR4A, we decided to investigate heterotypic membrane fusion (i.e., the merger between two membranes of distinct composition). For that, we prepared SLBs made of DOPC, DOPE and DOPS (4:3:3 mol ratio), and incubated them with GUVs made of POPC, and cholesterol (2:1 mol ratio). We simultaneously labeled the SLB and GUVs by adding 500 nM NR4A. Both membrane environments could be distinguished by their different lipid packing in confocal measurements, as reported by the GP parameter (Fig. 5 *A*, *top left panel*). Subsequent addition of 10 mM calcium chloride readily promoted membrane fusion (Fig. 5 *A*; Video S5), in line with the well-characterized fusogenic activity of divalent cations (62). By monitoring the process in real time, we could differentiate the following steps (Video S5): 1) contact, the vesicle is brought in close contact to the supported bilayer, the GUV membrane is highly mobile; 2) hemifusion, the vesicle is docked, lipid exchange can be observed as reported by the GP change (Fig. 5 *B*); and 3) full fusion, both membranes form a continuous bilayer. Because NR4A only partitions to the outer leaflet of bilayers, complete GP equilibrium could be observed after hemifusion.

Endo- and exocytosis involve significantly smaller vesicles than GUVs. Therefore, we next investigated the fusion of submicrometric-sized vesicles with SLBs, which could be resolved by 3D-STED GP imaging (Fig. 5 *C*; Video S6). Lipid exchange and fusion happened in a shorter timescale (Fig. 5 *D*) because of the smaller size of the vesicles. Finally, we prepared 400-nm LUVs, smaller than the axial resolution of confocal microscopy (Fig. S5, *A and B*; Video S7). In this case, fusion occurred seconds after attachment, and total lipid exchange occurred within two frames (Fig. S5, *C*–*E*), which could only be resolved with 3D-STED imaging. In summary, we show that exchangeable polarity-sensitive dyes can be useful tools for imaging fusion in real time with conventional or super-resolved fluorescence microscopy. Lipid exchange could be quantified by means of GP imaging, resolving the different steps of the fusion process.

DISCUSSION

The increase of spatial resolution to the subdiffraction scale by SRM is usually accompanied by a decrease in temporal resolution. In the case of STED SRM, photobleaching induced by the depletion laser hampers long-term experiments, especially when using the high laser powers needed to achieve the highest resolution. Here, we studied whether exchangeable polarity-sensitive probes allow the long-term quantification of two important membrane biophysical properties, namely lipid packing and membrane dynamics. To this end, we selected NR4A, a Nile-red-based probe designed for selective imaging of plasma membranes using SMLM (12). Nile red presents an intermediate affinity for membranes, promoting its continuous exchange between solution and membrane environments. This phenomenon was recently exploited to prevent photobleaching-induced signal loss in STED biomembrane imaging (36). However, Nile red nonspecifically labels all cell membranes, showing no selectivity and hampering the accurate measurement of

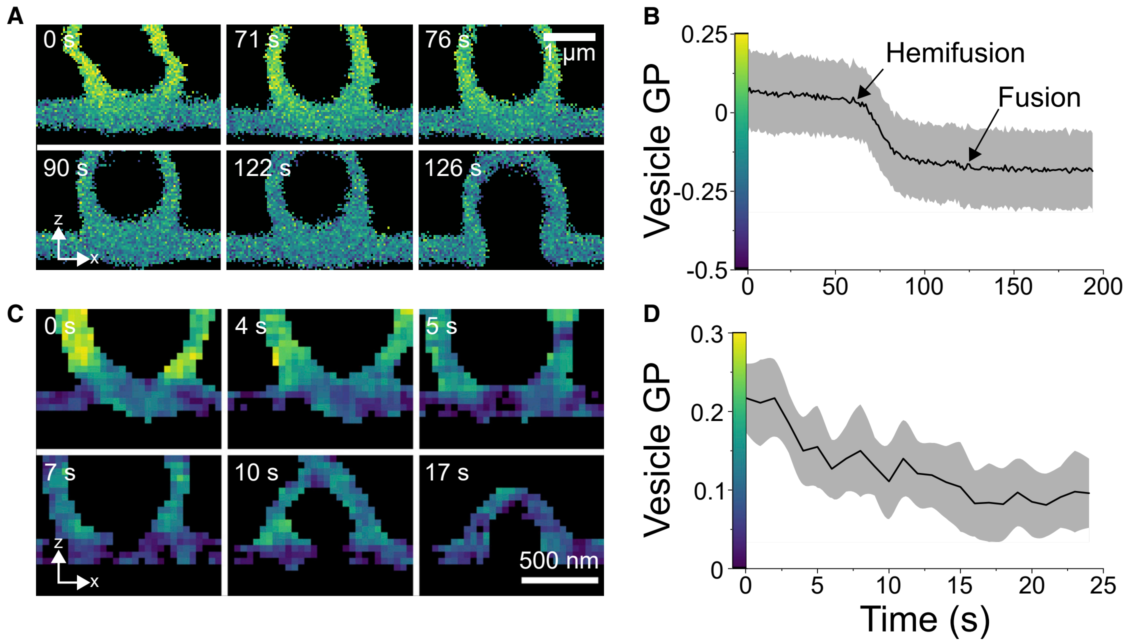

FIGURE 5 Real-time visualization of membrane fusion by confocal and 3D-STED microscopy of NR4A. (*A*) *xz* GP micrographs of a confocal time-lapse of a GUV made of DOPC:DOPE:DOPS (4:3:3 mol ratio) and a POPC:Chol (2:1 mol ratio) SLB. 10 mM calcium addition promoted membrane fusion that occurred after hemifusion and lipid exchange. GP changes of the GUV over time are shown in (*B*). (*C*) Membrane fusion and lipid packing monitoring of a submicrometer-sized vesicle. Lipid compositions are as in (*A*). (*D*) Vesicle lipid packing change over time. Scale bars, 1 μm in (*A*) and 500 nm in (*C*), as indicated. In (*B* and *D*), the grayed area is the SD of the vesicle GP in (*A*) and (*C*), respectively. All measurements were performed at room temperature.

spectral changes necessary for packing quantification of specific organelles. The sulfonate group in NR4A reduces its flip-flop rate (12,45) and thus ensures specific plasma membrane labeling for prolonged periods of time (Fig. 2, *C and D*; Video S2). Importantly, thanks to its exchangeable nature, NR4A can circumvent signal loss induced by dye photobleaching, even at the highest depletion laser powers used in STED microscopy (Fig. 2, *A and B*; Video S1). Thus, NR4A can be used for long-term imaging of model and plasma membranes, opening new opportunities for monitoring membrane biophysical properties in long-term experiments, as exemplified by our measurements of membrane fusion (Fig. 5; Videos S5, S6, and S7) and plasma membrane vesiculation (Fig. 4 *C*; Video S4).

The compatibility of STED microscopy with spectroscopic measurements permits the quantification of membrane biophysical properties, such as lipid diffusion by STED-FCS (5) or membrane packing with polarity-sensitive dyes (16). To obtain the highest spatial and temporal resolutions in STED microscopy, fluorescent dyes must offer high molecular brightness to ensure high signal/noise ratio. This enables the use of higher scanning frequencies, reducing photobleaching and phototoxicity (27), and permits shorter acquisitions in the case of STED-FCS. Here, we show that NR4A offers a high molecular brightness (Fig. 3 *B*) that yields higher resolution (Fig. 3 *A*), and less noisy STED-FCS measurements (Fig. 3 *C*). We employed STED-FCS to simultaneously measure membrane packing and dynamics, which were found to strongly anticorrelate in model and plasma membranes (Fig. 3 *D*). Importantly, we found that, despite its exchangeable nature, NR4A's emission spectra reports on membrane packing changes with high sensitivity in model (Fig. 1, *C and D*) and live-cell plasma membranes (Fig. 1, *E–G*; Fig. 4 *A*).

In conclusion, NR4A can circumvent signal loss induced by dye photobleaching, especially in STED microscopy. NR4A is sensitive to changes on lipid composition in confocal (Fig. 1) and STED (Fig. 4) microscopy. It permits continuous STED imaging of plasma membranes, which is no longer limited by photobleaching but rather by cell photodamage (Fig. 2). Signal fluctuation analysis methods, such as FCS or its super-resolved STED-FCS mode, can simultaneously report on packing and dynamics in model and plasma membranes (Fig. 3). NR4A is compatible with 3D-STED, which offers an order-of-magnitude increase in resolution in all axes, permitting the long-time visualization of previously unresolvable processes in cells (Fig. 4) and model membrane systems (Fig. 5). Finally, we would like to highlight the versatility of NR4A, which can be used in confocal, widefield, SMLM (12), and STED microscopy experiments.

Limitations of this study

Here, NR4A is presented as a STEDable dye that can circumvent photobleaching-induced signal loss. However, STED imaging of plasma and model membranes still presents several obstacles that need to be addressed before attaining the ultimate goal of unlimited live imaging with molecular resolution. First, the resolution offered by NR4A and NR12A is around 80 nm, approximately half of the resolution commonly achieved with live-cell STED microscopy of fluorescent lipid analogs labeled with dyes such as Abberior STAR RED or ATTO647N. Still, to the best of our knowledge, NR4A and NR12A are the polarity-sensitive dyes offering the best STED resolution so far. Moreover, to achieve the highest resolution, STED microscopy requires the use of highly focused laser beams with a power in the range of hundreds of milliwatts, which can induce cell photodamage (63), hampering long-time live-cell studies.

Polarity-sensitive dyes strongly suffer from photoselection (i.e., the preferential excitation of the fluorophore in respect to the excitation light polarization), which can introduce an underestimation in lipid packing quantification. Because membranes fix the orientation of polarity-sensitive dyes, *xy* imaging needs to be performed with circularly polarized light, whereas 3D/*z* imaging would require a combination of circularly and axially polarized light, an approach that, to the best of our knowledge, remains unexplored. Furthermore, Nile-red-based dyes show excitation and emission spectra that overlap with those of the most popular fluorophores and fluorescent proteins, hampering their combined use. The ability to transiently bind to their target structure confers exchangeable dyes their switchable nature; however, because most of the dye remains in solution, the concentrations needed to perform each experiment are at least one order of magnitude higher than that with classic membrane dyes, significantly increasing their cost. Moreover, when used at high concentration (500 nM), NR4A internalization can also impose a limitation if processes that span for longer than an hour are imaged.

SUPPORTING MATERIAL

Supporting material can be found online at https://doi.org/10.1016/j.bpr.2021.100023.

AUTHOR CONTRIBUTIONS

P.C. and E.S. did the experimental design. P.C., A.D., and G.Z. performed the data analysis. P.C., A.D., G.Z., and E.S. did the experiments. D.I.D. and A.S.K. performed dye synthesis. E.S. and C.E. supervised the project. C.E. was in charge of funding acquisition. The manuscript was written by P.C., with contributions from all authors. All authors have given approval to the final version of the manuscript.

ACKNOWLEDGMENTS

We thank the Microverse Imaging Center (Jena, Germany) (Aurélie Jost) for providing microscope facility support. We thank National Microscopy Infrastructure Sweden and Advanced Light Microscopy Facility at SciLifeLab (Solna, Sweeden) for their support on microscopy imaging.

P.C. received funding from the European Commission Horizon 2020 Marie Skłodowska Curie Programme (H2020-MSCA-IF-2019-ST project 892232 FILM-HIV) and the Basque Government (POS_2018_1_0066 and POS_2019_2_0022). We further acknowledge funding by the Deutsche Forschungsgemeinschaft (German Research Foundation; Germany's Excellence Strategy EXC 2051, project identifier 390713860; project number 316213987 – SFB 1278; Multi-User Equipment Grant: Two-photon stimulated emission depletion microscopy of membrane), State of Thuringia (TAB)-EFRE funding (Research Group SARS-Rapid; Advanced stimulated emission depletion microscopy), the Medical Research Council (grant No. MC_UU_12010/unit programs G0902418 and MC_UU_12025), the EPA Cephalosporin Fund, and the John Fell Fund. The Abberior Expert Line setup was funded by Deutsche Forschungsgemeinschaft grant INST 1757/25-1 FUGG and the State of Thuringia (TMWWDG). E.S. is supported by SciLifeLab, the Knut and Alice Wallengberg Foundation, the Karolinska Institutet, and the Swedish Research Council.

DECLARATION OF INTERESTS

SATT Conectus on behalf of University of Strasbourg and CNRS (A.S.K.) has a Material Transfer and Data License Agreement with Cytoskeleton, Inc., concerning NR12A and NR4A. The rest of the authors declare no competing interests.

REFERENCES

1. Sezgin, E., I. Levental, …, C. Eggeling. 2017. The mystery of membrane organization: composition, regulation and roles of lipid rafts. *Nat. Rev. Mol. Cell Biol.* 18:361–374.
2. Levental, K. R., M. A. Surma, …, I. Levental. 2017. ω-3 polyunsaturated fatty acids direct differentiation of the membrane phenotype in mesenchymal stem cells to potentiate osteogenesis. *Sci. Adv.* 3:eaao1193.
3. Favard, C., J. Chojnacki, …, D. Muriaux. 2019. HIV-1 Gag specifically restricts PI(4,5)P2 and cholesterol mobility in living cells creating a nanodomain platform for virus assembly. *Sci. Adv.* 5:eaaw8651.
4. Sengupta, P., A. Y. Seo, …, J. Lippincott-Schwartz. 2019. A lipid-based partitioning mechanism for selective incorporation of proteins into membranes of HIV particles. *Nat. Cell Biol.* 21:452–461.
5. Eggeling, C., C. Ringemann, …, S. W. Hell. 2009. Direct observation of the nanoscale dynamics of membrane lipids in a living cell. *Nature.* 457:1159–1162.
6. Schwille, P., J. Korlach, and W. W. Webb. 1999. Fluorescence correlation spectroscopy with single-molecule sensitivity on cell and model membranes. *Cytometry.* 36:176–182.
7. Yu, W., P. T. So, …, E. Gratton. 1996. Fluorescence generalized polarization of cell membranes: a two-photon scanning microscopy approach. *Biophys. J.* 70:626–636.

8. Colom, A., E. Derivery, …, A. Roux. 2018. A fluorescent membrane tension probe. *Nat. Chem.* 10:1118–1125.
9. Sezgin, E. 2017. Super-resolution optical microscopy for studying membrane structure and dynamics. *J. Phys. Condens. Matter.* 29:273001.
10. Betzig, E., G. H. Patterson, …, H. F. Hess. 2006. Imaging intracellular fluorescent proteins at nanometer resolution. *Science.* 313:1642–1645.
11. Balzarotti, F., Y. Eilers, …, S. W. Hell. 2017. Nanometer resolution imaging and tracking of fluorescent molecules with minimal photon fluxes. *Science.* 355:606–612.
12. Danylchuk, D. I., S. Moon, …, A. S. Klymchenko. 2019. Tailor-made switchable solvatochromic probes for live-cell super-resolution imaging of plasma membrane organization. *Angew. Chem.* 131:15062–15066.
13. Núñez, M. F., K. Wisser, and S. L. Veatch. 2020. Synergistic factors control kinase-phosphatase organization in B-cells engaged with supported bilayers. *Mol. Biol. Cell.* 31:667–682.
14. Vicidomini, G., P. Bianchini, and A. Diaspro. 2018. STED super-resolved microscopy. *Nat. Methods.* 15:173–182.
15. Hedde, P. N., R. M. Dörlich, …, G. U. Nienhaus. 2013. Stimulated emission depletion-based raster image correlation spectroscopy reveals biomolecular dynamics in live cells. *Nat. Commun.* 4:2093.
16. Sezgin, E., F. Schneider, …, C. Eggeling. 2017. Polarity-sensitive probes for superresolution stimulated emission depletion microscopy. *Biophys. J.* 113:1321–1330.
17. Barbotin, A., I. Urbančič, …, E. Sezgin. 2020. z-STED imaging and spectroscopy to investigate nanoscale membrane structure and dynamics. *Biophys. J.* 118:2448–2457.
18. Eggeling, C., K. I. Willig, …, S. W. Hell. 2015. Lens-based fluorescence nanoscopy. *Q. Rev. Biophys.* 48:178–243.
19. Donnert, G., J. Keller, …, S. W. Hell. 2006. Macromolecular-scale resolution in biological fluorescence microscopy. *Proc. Natl. Acad. Sci. USA.* 103:11440–11445.
20. Oracz, J., V. Westphal, …, S. W. Hell. 2017. Photobleaching in STED nanoscopy and its dependence on the photon flux applied for reversible silencing of the fluorophore. *Sci. Rep.* 7:11354.
21. Song, L., C. A. Varma, …, H. J. Tanke. 1996. Influence of the triplet excited state on the photobleaching kinetics of fluorescein in microscopy. *Biophys. J.* 70:2959–2968.
22. Eggeling, C., J. Widengren, …, C. A. Seidel. 1998. Photobleaching of fluorescent dyes under conditions used for single-molecule detection: evidence of two-step photolysis. *Anal. Chem.* 70:2651–2659.
23. Hotta, J., E. Fron, …, J. Hofkens. 2010. Spectroscopic rationale for efficient stimulated-emission depletion microscopy fluorophores. *J. Am. Chem. Soc.* 132:5021–5023.
24. Kolmakov, K., V. N. Belov, …, S. W. Hell. 2010. Red-emitting rhodamine dyes for fluorescence microscopy and nanoscopy. *Chemistry.* 16:158–166.
25. Zheng, Q., and L. D. Lavis. 2017. Development of photostable fluorophores for molecular imaging. *Curr. Opin. Chem. Biol.* 39:32–38.
26. Donnert, G., C. Eggeling, and S. W. Hell. 2007. Major signal increase in fluorescence microscopy through dark-state relaxation. *Nat. Methods.* 4:81–86.
27. Wu, Y., X. Wu, …, E. Stefani. 2015. Resonant scanning with large field of view reduces photobleaching and enhances fluorescence yield in STED microscopy. *Sci. Rep.* 5:14766.
28. Donnert, G., C. Eggeling, and S. W. Hell. 2009. Triplet-relaxation microscopy with bunched pulsed excitation. *Photochem. Photobiol. Sci.* 8:481–485.
29. Heine, J., M. Reuss, …, S. W. Hell. 2017. Adaptive-illumination STED nanoscopy. *Proc. Natl. Acad. Sci. USA.* 114:9797–9802.
30. Sharonov, A., and R. M. Hochstrasser. 2006. Wide-field subdiffraction imaging by accumulated binding of diffusing probes. *Proc. Natl. Acad. Sci. USA.* 103:18911–18916.
31. Jungmann, R., M. S. Avendaño, …, P. Yin. 2014. Multiplexed 3D cellular super-resolution imaging with DNA-PAINT and Exchange-PAINT. *Nat. Methods.* 11:313–318.
32. Gao, F., E. Mei, …, R. M. Hochstrasser. 2006. Probing lipid vesicles by bimolecular association and dissociation trajectories of single molecules. *J. Am. Chem. Soc.* 128:4814–4822.
33. Bongiovanni, M. N., J. Godet, …, S. F. Lee. 2016. Multi-dimensional super-resolution imaging enables surface hydrophobicity mapping. *Nat. Commun.* 7:13544.
34. Moon, S., R. Yan, …, K. Xu. 2017. Spectrally resolved, functional super-resolution microscopy reveals nanoscale compositional heterogeneity in live-cell membranes. *J. Am. Chem. Soc.* 139:10944–10947.
35. Yan, R., K. Chen, and K. Xu. 2020. Probing nanoscale diffusional heterogeneities in cellular membranes through multidimensional single-molecule and super-resolution microscopy. *J. Am. Chem. Soc.* 142:18866–18873.
36. Spahn, C., J. B. Grimm, …, M. Heilemann. 2019. Whole-cell, 3D, and multicolor STED imaging with exchangeable fluorophores. *Nano Lett.* 19:500–505.
37. Sackett, D. L., J. R. Knutson, and J. Wolff. 1990. Hydrophobic surfaces of tubulin probed by time-resolved and steady-state fluorescence of nile red. *J. Biol. Chem.* 265:14899–14906.
38. Hou, Y., A. M. Bardo, …, D. A. Higgins. 2000. Characterization of molecular scale environments in polymer films by single molecule spectroscopy. *J. Phys. Chem. B.* 104:212–219.
39. Reichardt, C. 1994. Solvatochromic dyes as solvent polarity indicators. *Chem. Rev.* 94:2319–2358.
40. Klymchenko, A. S. 2017. Solvatochromic and fluorogenic dyes as environment-sensitive probes: design and biological applications. *Acc. Chem. Res.* 50:366–375.
41. Parasassi, T., G. De Stasio, …, E. Gratton. 1990. Phase fluctuation in phospholipid membranes revealed by Laurdan fluorescence. *Biophys. J.* 57:1179–1186.
42. Klymchenko, A. S., and R. Kreder. 2014. Fluorescent probes for lipid rafts: from model membranes to living cells. *Chem. Biol.* 21:97–113.
43. Jin, L., A. C. Millard, …, L. M. Loew. 2005. Cholesterol-enriched lipid domains can be visualized by di-4-ANEPPDHQ with linear and nonlinear optics. *Biophys. J.* 89:L04–L06.
44. Jin, L., A. C. Millard, …, L. M. Loew. 2006. Characterization and application of a new optical probe for membrane lipid domains. *Biophys. J.* 90:2563–2575.
45. Danylchuk, D. I., E. Sezgin, …, A. S. Klymchenko. 2020. Redesigning solvatochromic probe laurdan for imaging lipid order selectively in cell plasma membranes. *Anal. Chem.* 92:14798–14805.
46. Kucherak, O. A., S. Oncul, …, A. S. Klymchenko. 2010. Switchable nile red-based probe for cholesterol and lipid order at the outer leaflet of biomembranes. *J. Am. Chem. Soc.* 132:4907–4916.
47. Owen, D. M., C. Rentero, …, K. Gaus. 2011. Quantitative imaging of membrane lipid order in cells and organisms. *Nat. Protoc.* 7:24–35.
48. Sezgin, E., D. Waithe, …, C. Eggeling. 2015. Spectral imaging to measure heterogeneity in membrane lipid packing. *ChemPhysChem.* 16:1387–1394.
49. Sezgin, E., F. Schneider, …, C. Eggeling. 2019. Measuring nanoscale diffusion dynamics in cellular membranes with super-resolution STED-FCS. *Nat. Protoc.* 14:1054–1083.
50. Schindelin, J., I. Arganda-Carreras, …, A. Cardona. 2012. Fiji: an open-source platform for biological-image analysis. *Nat. Methods.* 9:676–682.

51. Lord, S. J., K. B. Velle, …, L. K. Fritz-Laylin. 2020. SuperPlots: communicating reproducibility and variability in cell biology. *J. Cell Biol.* 219:e202001064.

52. Sezgin, E., H.-J. Kaiser, …, I. Levental. 2012. Elucidating membrane structure and protein behavior using giant plasma membrane vesicles. *Nat. Protoc.* 7:1042–1051.

53. Waithe, D., M. P. Clausen, …, C. Eggeling. 2016. FoCuS-point: software for STED fluorescence correlation and time-gated single photon counting. *Bioinformatics.* 32:958–960.

54. Eggeling, C., A. Volkmer, and C. A. M. Seidel. 2005. Molecular photobleaching kinetics of Rhodamine 6G by one- and two-photon induced confocal fluorescence microscopy. *ChemPhysChem.* 6:791–804.

55. Carravilla, P., J. L. Nieva, …, N. Huarte. 2015. Two-photon Laurdan studies of the ternary lipid mixture DOPC:SM:cholesterol reveal a single liquid phase at sphingomyelin:cholesterol ratios lower than 1. *Langmuir.* 31:2808–2817.

56. van Meer, G., D. R. Voelker, and G. W. Feigenson. 2008. Membrane lipids: where they are and how they behave. *Nat. Rev. Mol. Cell Biol.* 9:112–124.

57. Sanchez, S. A., M. A. Tricerri, and E. Gratton. 2012. Laurdan generalized polarization fluctuations measures membrane packing micro-heterogeneity in vivo. *Proc. Natl. Acad. Sci. USA.* 109:7314–7319.

58. Bernabé-Rubio, M., M. Bosch-Fortea, …, M. A. Alonso. 2021. Adaptive lipid immiscibility and membrane remodeling are active functional determinants of primary ciliogenesis. *Small Methods.* 5:2000711.

59. Urbančič, I., J. Brun, …, J. Chojnacki. 2018. Lipid composition but not curvature is the determinant factor for the low molecular mobility observed on the membrane of virus-like vesicles. *Viruses.* 10:E415.

60. Klar, T. A., S. Jakobs, …, S. W. Hell. 2000. Fluorescence microscopy with diffraction resolution barrier broken by stimulated emission. *Proc. Natl. Acad. Sci. USA.* 97:8206–8210.

61. Skinkle, A. D., K. R. Levental, and I. Levental. 2020. Cell-derived plasma membrane vesicles are permeable to hydrophilic macromolecules. *Biophys. J.* 118:1292–1300.

62. Wilschut, J., N. Düzgüneş, and D. Papahadjopoulos. 1981. Calcium/magnesium specificity in membrane fusion: kinetics of aggregation and fusion of phosphatidylserine vesicles and the role of bilayer curvature. *Biochemistry.* 20:3126–3133.

63. Kilian, N., A. Goryaynov, …, J. Bewersdorf. 2018. Assessing photodamage in live-cell STED microscopy. *Nat. Methods.* 15:755–756.

Biophysical Reports, Volume *1*

Supplemental information

Long-term STED imaging of membrane packing and dynamics by exchangeable polarity-sensitive dyes

Pablo Carravilla, Anindita Dasgupta, Gaukhar Zhurgenbayeva, Dmytro I. Danylchuk, Andrey S. Klymchenko, Erdinc Sezgin, and Christian Eggeling

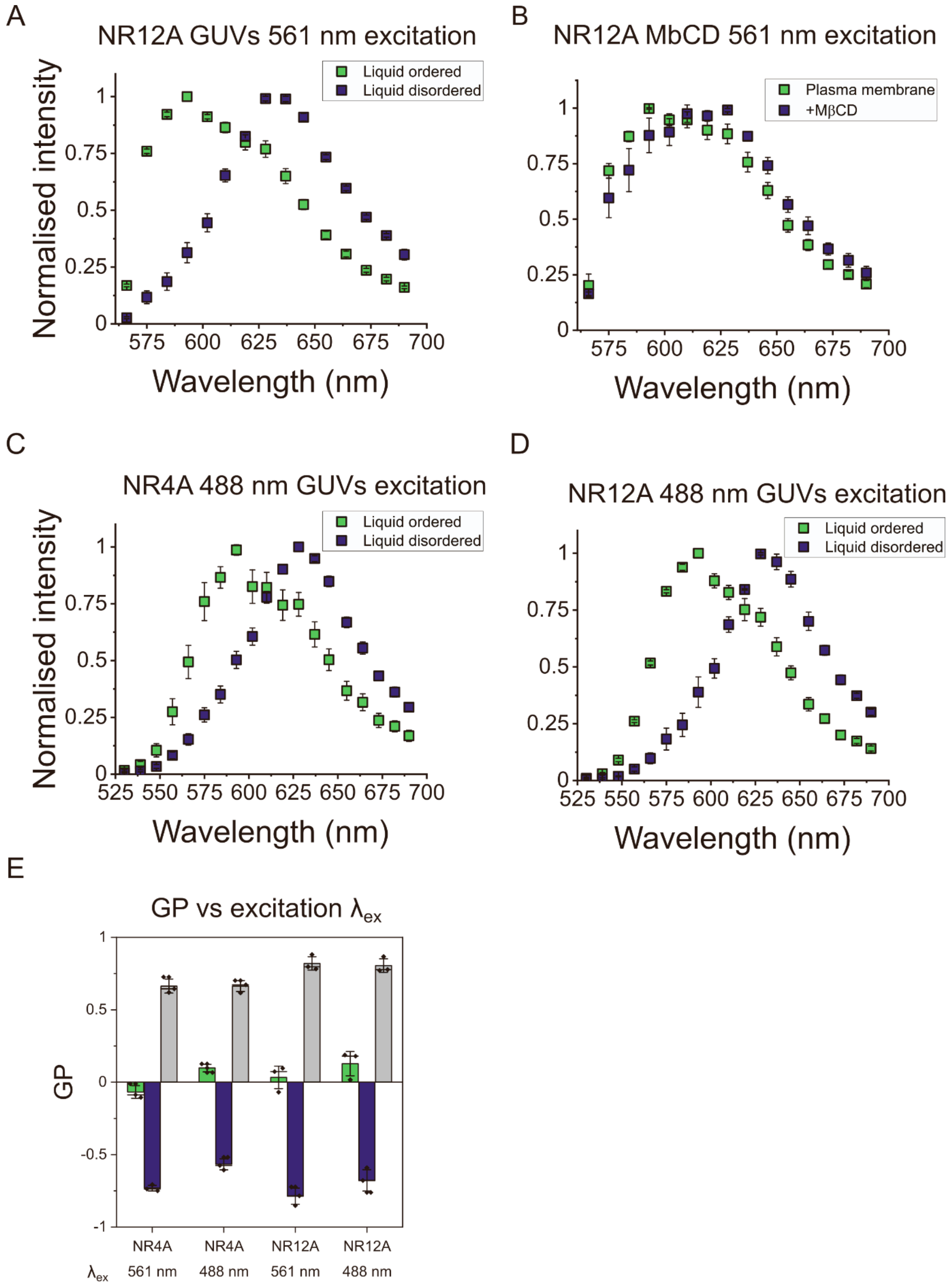

Supplementary Figure 1. Characterisation of NR4A and NR12A spectral properties. (A) Emission spectra of NR12A in phase separated GUVs. Conditions as in Figure 1C. (B) Emission spectra of NR12A in control and MβCD-treated C2BBe1 cells. Conditions as in Figure 1E. (C) and (D) emission spectra of NR4A and NR12A, respectively in phase-separated GUVs under 488 nm excitation. 561 nm excitation was preferred since it yielded a higher intensity signal (data not shown). Conditions as in Figure 1C. (E) GP-resolution comparison between 488 nm and 561 nm excitation of NR4A and NR12A in phase-separated GUVs. Conditions as in Figure 1D.

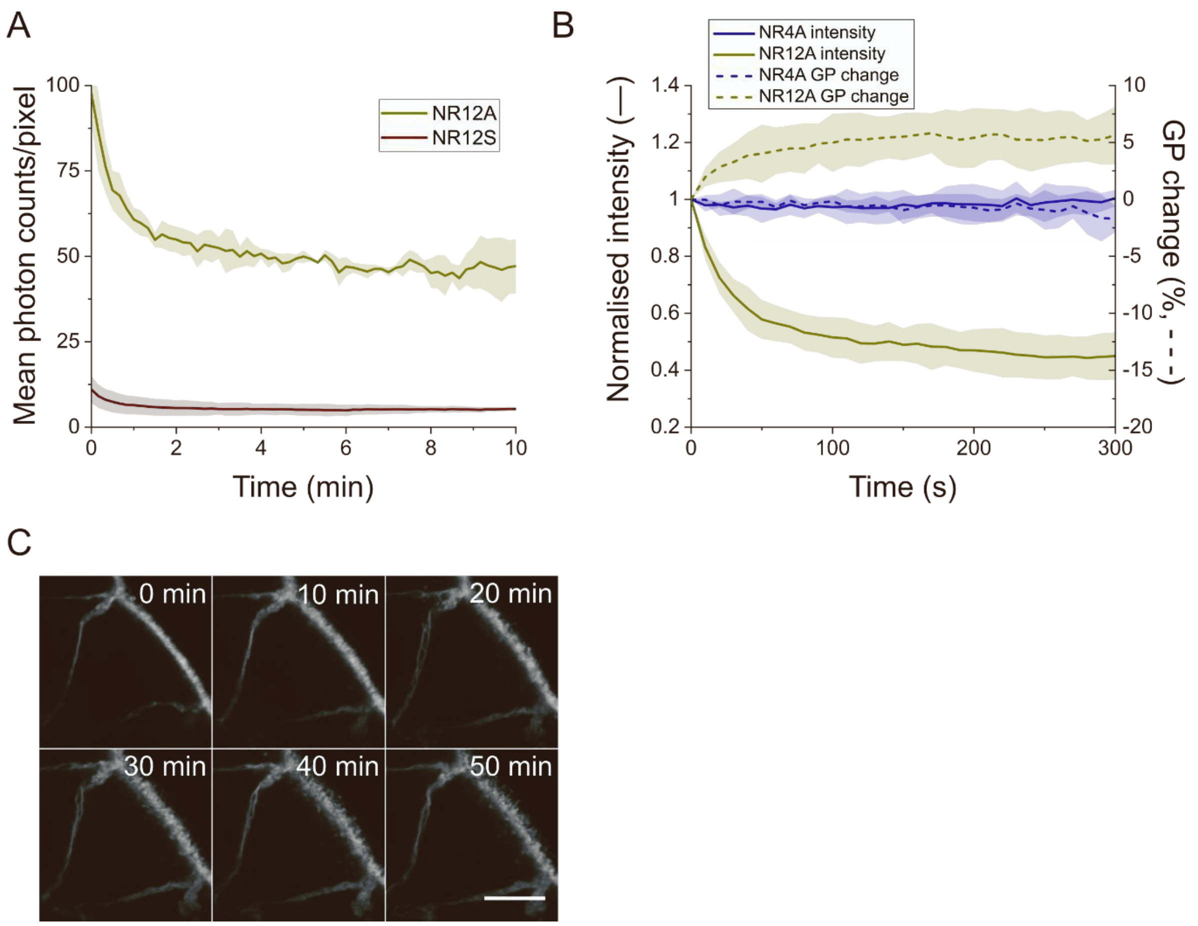

Supplementary Figure 2. Plasma membrane STED imaging of polarity-sensitive dyes NR4A, NR12A and NR12S. (A) Raw intensity signal comparison of Ptk2 plasma membrane signal between NR12S, a previous generation Nile Red derivative, and NR12A. (B) STED laser-induced photobleaching causes a ca. 5% change in the measured GP value in Ptk2 plasma membranes. (C) Micrographs of confocal images of C2BBe1 cells labelled with NR12A to measure dye internalisation. Internalisation quantification is shown in Figure 2D. Otherwise, conditions as in Figure 2.

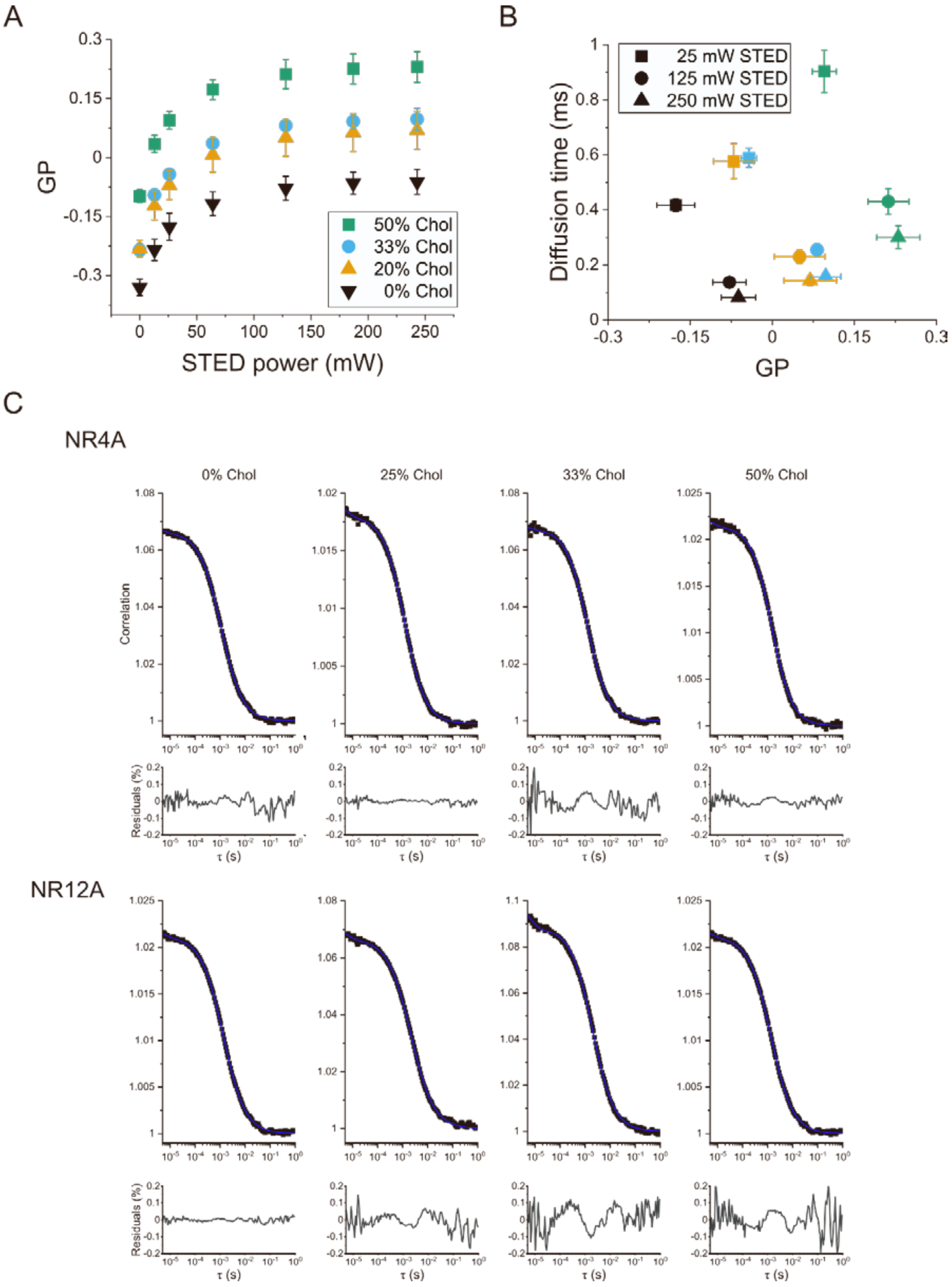

Supplementary Figure 3. STED-FCS measurements of NR4A. (A) NR4A GP values measured in cholesterol containing SLBs. GP values depend on the STED laser power but show a high sensitivity at all measured powers. (B) Dynamics and lipid packing correlate independently of the STED laser power used. Colour codes as in panel A. Conditions as in Figure 3D. (C) Exemplary FCS curves (black symbols), fitting to 2D Brownian model (see Materials and Methods, blue lines), and fitting residuals (% of maximum amplitude of the curve, grey lines), for NR4A (top) and NR12A (bottom), as measured on SLBs with different cholesterol contents (0%, 25%, 33% and 50%, from left to right). Fixed fitting parameters were triplet time (5 μs), alpha (1). G(0), diffusion time and offset were left floating.

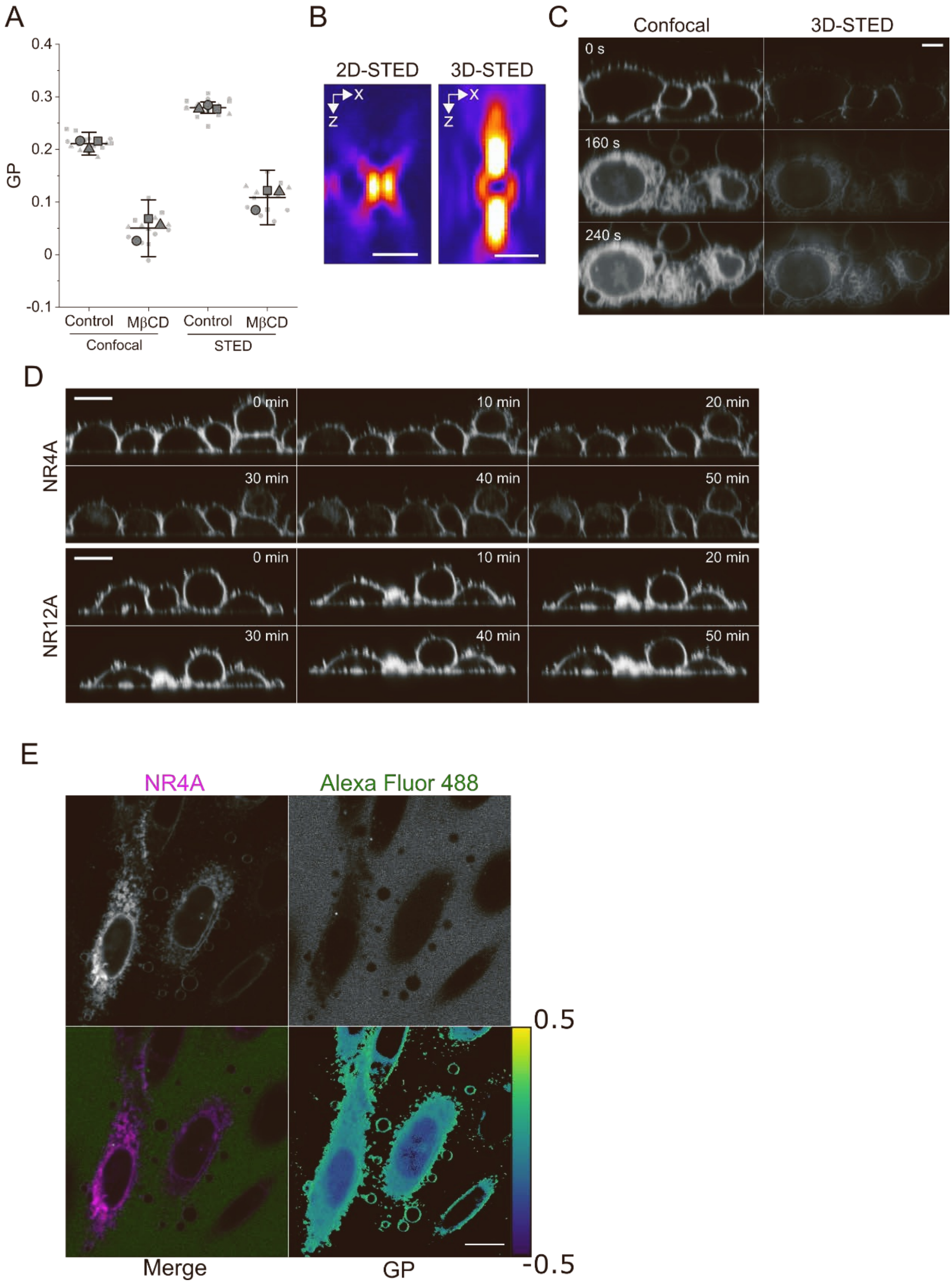

Supplementary Figure 4. NR4A imaging of live cell plasma membranes. (A) Comparison between confocal and STED-GP values of control and MβCD-treated (20 mM, 1h, 37 °C) C2BBe1 cells. STED laser power was 250 mW. Conditions as in Figure 4A. (B) Depletion patterns for 2D- and 3D-STED as imaged on scattering 150 nm gold nanoparticles. Scale bar is 1 µm. (C) xz view of plasma membrane vesiculation of 293T/17 cells induced by DTT/PFA treatment at 37 °C. Conditions as in Figure 4C. Scale bar is 5 µm. (D) Micrographs of confocal *xz* images of the internalisation of NR4A (top) and NR12A (bottom) in CHO cells. Scale bar is 10 µm. (E) Micrographs of CHO cells after 30-minute DTT/PFA treatment. DTT/PFA induce internalisation of NR4A (magenta, 500 nM), but not of the small hydrophilic Alexa Fluor 488 (green, 1 µM). GP images shows the lower packing of internal cell membranes.

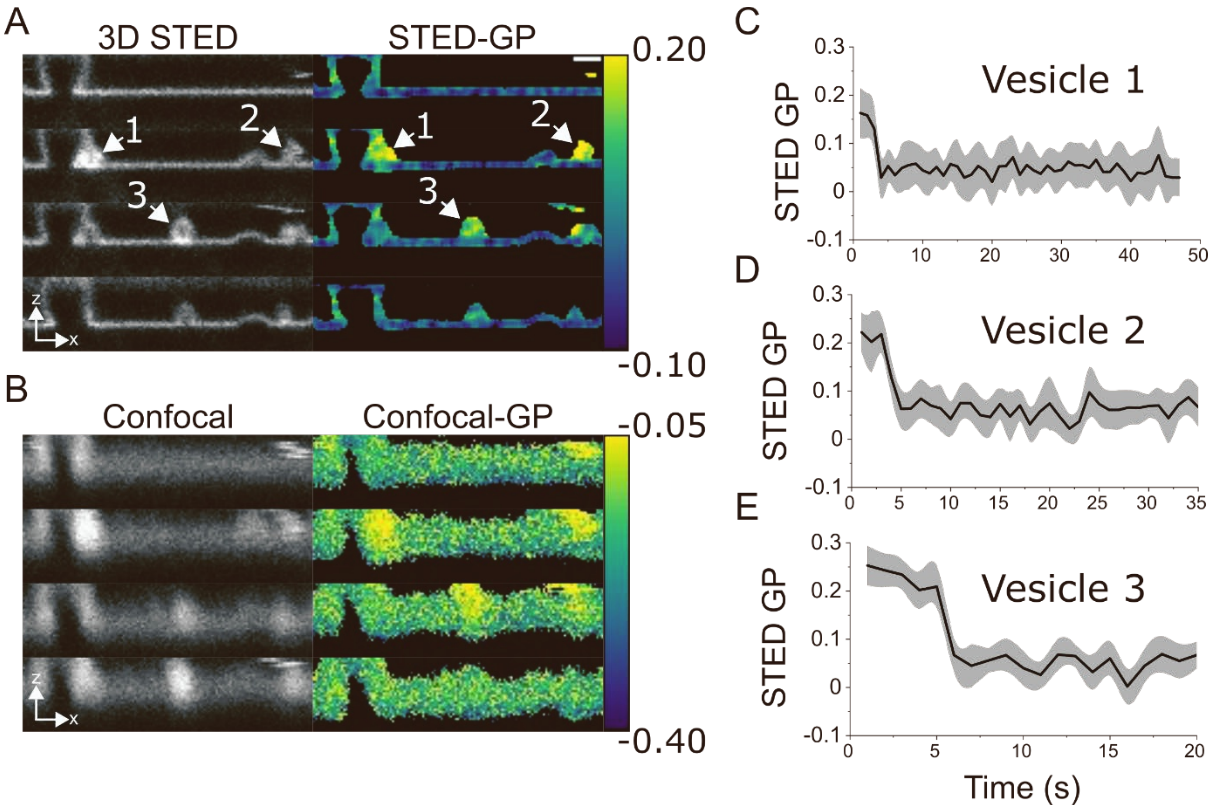

Supplementary Figure 5. Real time visualisation of LUV fusion with an SLB. (A) 3D-STED intensity (left) and GP (right) images of POPC:cholesterol (2:1 mol ratio) LUVs fusing with a DOPC:DOPE:DOPS (4:3:3 mol ratio) bilayer. Scale bar is 500 nm. (B) Same as in A, but acquired in confocal mode. (C-E) Quantification of the GP changes over time of the vesicles marked with an arrowhead in panel A. Otherwise conditions as in Figure 5.
